# The clinical usefulness of optical coherence tomography during cancer interventions

**DOI:** 10.1007/s00432-018-2690-9

**Published:** 2018-06-20

**Authors:** Labrinus van Manen, Jouke Dijkstra, Claude Boccara, Emilie Benoit, Alexander L. Vahrmeijer, Michalina J. Gora, J. Sven D. Mieog

**Affiliations:** 1 Department of Surgery, Leiden University Medical Center, Albinusdreef 2, 2300 RC Leiden, The Netherlands; 2 Division of Image Processing, Department of Radiology, Leiden University Medical Center, Leiden, The Netherlands; 3 Institut Langevin, Paris, France; 4 LLTech, Paris, France; 5 ICube Laboratory, CNRS, Strasbourg University, Strasbourg, France

**Keywords:** Optical coherence tomography, Cancer, Tumor, Image-guided surgery, Optical imaging.

## Abstract

**Introduction:**

Tumor detection and visualization plays a key role in the clinical workflow of a patient with suspected cancer, both in the diagnosis and treatment. Several optical imaging techniques have been evaluated for guidance during oncological interventions. Optical coherence tomography (OCT) is a technique which has been widely evaluated during the past decades. This review aims to determine the clinical usefulness of OCT during cancer interventions focussing on qualitative features, quantitative features and the diagnostic value of OCT.

**Methods:**

A systematic literature search was performed for articles published before May 2018 using OCT in the field of surgical oncology. Based on these articles, an overview of the clinical usefulness of OCT was provided per tumor type.

**Results:**

A total of 785 articles were revealed by our search, of which a total of 136 original articles were available for analysis, which formed the basis of this review. OCT is currently utilised for both preoperative diagnosis and intraoperative detection of skin, oral, lung, breast, hepatobiliary, gastrointestinal, urological, and gynaecological malignancies. It showed promising results in tumor detection on a microscopic level, especially using higher resolution imaging techniques, such as high-definition OCT and full-field OCT.

**Conclusion:**

In the near future, OCT could be used as an additional tool during bronchoscopic or endoscopic interventions and could also be implemented in margin assessment during (laparoscopic) cancer surgery if a laparoscopic or handheld OCT device will be further developed to make routine clinical use possible.

## Introduction

Tumor detection and visualization plays a key role in the clinical workflow of a patient with suspected cancer, both in the diagnosis and in the treatment. During the last decades, numerous imaging modalities, such as ultrasound (US), computed tomography (CT), and magnetic resonance imaging (MRI), have proven additional value in establishing the diagnosis of an oncologic patient. Nevertheless, pathologic analysis of representative tumor biopsies is often necessary for establishing the correct diagnosis.

Furthermore, intraoperative detection of the tumor margins is difficult, as surgeons currently mainly rely on visualization and palpation. Pathological techniques to examine the margins intraoperatively, such as frozen section analysis and imprint cytology, have been extensively researched for the purpose of reducing the percentage of positive margins in breast cancer surgery, for instance. However, all these methods have drawbacks, such as time-consuming and resource-intensive nature, difficulty in visualizing high-grade carcinomas, and imprecision, due to sampling errors and poor resolution (Haka et al. 2006; Kennedy et al. 2010; Revesz and Khan 2011). With their ability to image molecular and physiological changes that are associated with cancer sensitively and non-invasively, optical imaging devices, such as optical coherence tomography (OCT), have the potential to improve intraoperative tumor detection (Frangioni 2008; Keereweer et al. 2011).

OCT is a technique that uses the interference of light to generate two-dimensional cross-sectional images. It was first described in 1991 and is often denoted as the optical analog of ultrasound; it detects back-reflected light, instead of sound, from tissues (Huang et al. 1991). In the field of cardiology and ophthalmology, it is already used as part of standard clinical care (Vakoc et al. 2012). OCT is, in contrast to other optical image modalities, able to image non-invasively and without the need for tissue preparation. The technique produces images, which are comparable to low-resolution histology. The resolution in comparison with US is 10–50 times better, and usually lies in the range of 1–20 µm in axial and transverse direction, depending on the modality used. This technique could be applied both for ex vivo and in vivo use. Moreover, in the last years, OCT was used during endoscopy or bronchoscopy, by incorporating OCT into flexible fiberoptic probes, which could be inserted in the accessory channel of the majority of standard of care scopes (Jung et al. 2004; Tearney et al. 1997a). For imaging with higher resolution and more cellular detail, high definition OCT (HD-OCT) and full-field OCT (FF-OCT) have been developed. HD-OCT is a commercially available system dedicated to skin imaging (Skintell^®^, Agfa Healthcare Mortsel, Belgium and München, Germany) providing axial and transversal resolution of 3 µm over 1.8 × 1.5 mm field of view, however, with penetration depth limited to 570 µm. The penetration depth is also limited to first few hundred microns in FF-OCT that directly acquires 2D en face images (without beam scanning) by illuminating the full field of view with a white-light source, such as a halogen lamp (Boone et al. 2012; Popescu et al. 2011). In FF-OCT, three-dimensional imaging can be performed, by stepping the reference mirror and recording successive en face images resulting in a stack of images (Dubois et al. 2004). With a speed limitation, which is caused by the long acquisition times, higher resolution OCT imaging can be only applied for ex vivo imaging.

The aim of this review is to determine the clinical usefulness of OCT and its variants during cancer interventions for both preoperative diagnosis and intraoperative tumor detection, with a focus on qualitative features, quantitative features and the diagnostic value of OCT, which are described per tumor type.

## Methods

A literature search in PubMed was performed for articles using OCT in the field of surgical oncology, published before May 2018. The search consisted of different keywords: “optical coherence tomography” or “OCT” combined with general terms (“oncology”, “oncologic”, “tumor”, “tumors”, “malignancy”, “malignancies”, and “cancer”) and more tumor-specific MeSH terms (“skin neoplasms”, “oral neoplasms”, “lung neoplasms”, “breast neoplasms”, “pancreatic neoplasms”, “liver neoplasms”, “bile duct neoplasms”, “esophageal neoplasms”, “stomach neoplasms”, “colorectal neoplasms”, “prostate neoplasms”, “kidney neoplasms”, “urinary bladder neoplasms”, and “ovarian neoplasms”). Case reports, (systematic) reviews, non-human studies, and articles not written in English were excluded from the analysis.

## Results

**Fig. 1:**
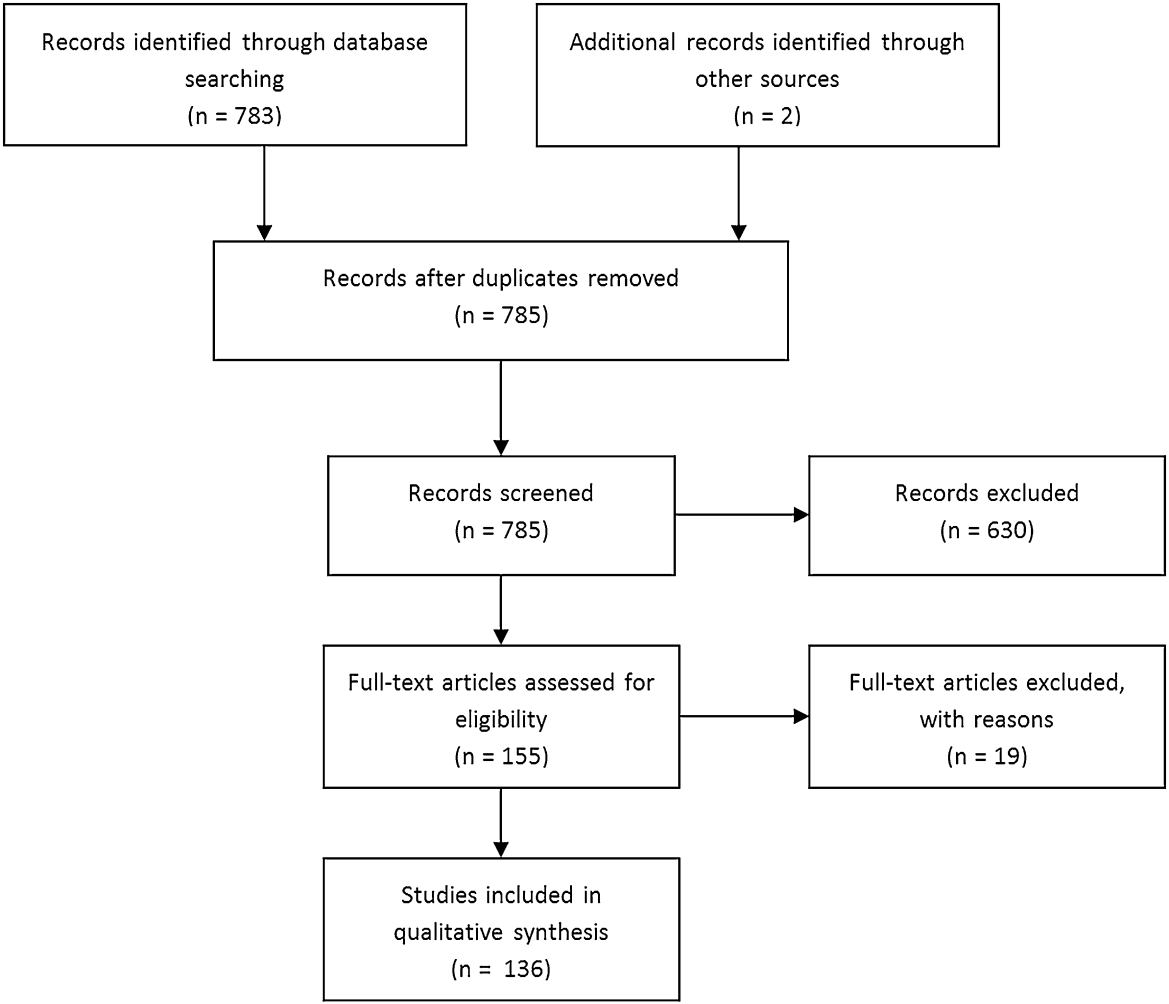
Flow diagram of study inclusion

A total of 785 articles were revealed by our search, of which two were found by manual search on the *SPIE digital library*. After exclusion of 649 articles, that did not meet our eligibility criteria, a total of 136 original articles remained, of which an overview is given in Fig. 1. The included articles, which form the basis of this review, are discussed separately per cancer type. An overview of the diagnostic value of OCT for tumor detection, including relevant study characteristics, is provided in Table 1.

**Table 1:** Overview of clinical studies evaluating the diagnostic value of optical coherence tomography for tumor detection

Tumor type	References	Technical specifications				Study design			Analysis	Diagnostic value		
		Technique, *Manufacturer*	Resolution: axial × lateral (µm)	Penetration depth (mm)	Acquisition time/image (s)	*N* (images)	Implementation	Samples		Sensitivity (%)	Specificity (%)	Detection rate (%)
Basal cell carcinoma	Mogensen et al. (2009a, b)	(Polarization-sensitive) OCT, *Technical University of Denmark*	8 × 24	NS	3	220	Ex vivo	Suspected lesions	6 reviewers of which 2 reviewed all images	79, 94	85, 96	–
	Jorgensen et al. (2008)	OCT, *Riso National Laboratory, Roskilde, Denmark*	10 × 20	1.3	4	78	Ex vivo	Suspected lesions	Machine based learning	–	–	81
	Ulrich et al. (2015)	OCT, *Vivosight Scanner, Michelson Diagnostics Ltd (Orpington, Kent, U.K.)*	5 × 7.5	1.2-2	NS	235	In vivo	Suspected lesions	Clinicians of participating centres	96	75	–
	Cunha et al. (2011)	OCT, *EX1301, Michelson Diagnostics Ltd (Orpington, Kent, U.K.)*	10 × 7.5	1.5	NS	75	Ex vivo	Resection margin	2 Mohs surgeons	19	56	–
	Maier et al. (2014)	HD-OCT, *Skintell, Agfa HealthCare, Belgium*	3 × 3	0.45–0.75	120	80	Ex vivo	Resection margin	1 Experienced investigator	74	64	–
Oral cancer	Wilder-Smith et al. (2009)	OCT, *Niris™ system, Imalux (Cleveland, OH)*	5–10 (not exactly specified)	1–2	1.5	50	Ex vivo	Biopsies	2 Reviewers	93	93	–
	Hamdoon et al. (2016)	OCT, *EX1301, Michelson Diagnostics Ltd (Orpington, Kent, U.K.)*	< 10 × < 10	1.5	< 0.1	112	Ex vivo	Resected SCC specimens	2 Reviewers	82	87	–
	De Leeuw et al. (2015)	FF-OCT, *Light CT scanner, LL-Tech SAS (Paris, France)*	1.5 × 1.0	NS	NS	57	Ex vivo	Resected head and neck specimens	2 Pathologists	88, 90	81, 87	–
Lung cancer	Hariri et al. (2015)	OCT, *Harvard Medical School (Boston, USA)*	6 × 30	2–3	NS	82	Ex vivo	Resection specimens	1 Pathologist 1 OCT expert 1 surgeon	80 (AC) 83 (SCC) 86 (PDC)	89 (AC) 87 (SCC) 98 (PDC)	–
Breast cancer	Nguyen et al. (2009)	OCT, *University of Illinois, Urbana-Champaign (Illinois, USA)*	6 × 35	1–2	5	210	Ex vivo	Resection margin	1 Trained researcher	100	82	–
	Zysk et al. (2015)	Handheld OCT, *University of Illinois, Urbana-Champaign (Illinois, USA)*	< 15 × < 15	NS	NS	2192	Ex vivo	Resection margin	1 Pathologist 1 surgeon 1 radiologist	55–65	68–70	–
	Erickson-Bhatt et al. (2015)	Handheld OCT, *University of Illinois, Urbana-Champaign (Illinois, USA)*	9 × 9	NS	NS	50	In vivo and ex vivo	Resection margin	5 Trained OCT readers	92	92	–
	Nolan et al. (2016)	OCT, *Bioptigen Inc. (Morrisville, USA)*	11 × 11	NS	300–600	184	Ex vivo	Lymph nodes	3 Analists	59	81	–
	Grieve et al. (2016)	FF-OCT, *LL-Tech SAS (Paris, France)*	1 × 1.6	0.20–0.30	600	71	Ex vivo	Lymph nodes	1 Pathologist 1 non-medical OCT expert	92/85	83/90	–
Pancreatico-biliary cancer	Testoni et al. (2005)	OCT, *Pentax, Lightlab Imaging (Westford, MA, USA)*	5–10 × 5–10	1	1 radial mm /s	100	Ex vivo	Resection specimens	3 Observers	79	89	–
	Testoni et al. (2007)	OCT, *Pentax, Lightlab Imaging (Westford, MA, USA)*	5–10 × 5–10	1	1 radial mm /s	11	In vivo (during ERCP)	Pancreatic duct strictures	NS	100	100	–
	Arvanitakis et al. (2009)	OCT, *PENTAX Corporation (Tokyo, Japan)* / *Lightlab Imaging Ltd. (Boston, USA)*	10 (not exactly described)	1	NS	35	In vivo (during ERCP)	Biliary duct strictures	2 Endoscopists	53	100	–
	Iftimia et al. (2011)	OCT, *Physical Sciences, Inc. (Andover, USA)*	9.5 × 25	NS	NS	46	Ex vivo	Resected cysts	1 Pathologist 1 gastroenterologist 1 radiologist	95	95	–
	Van Manen et al. (2017)	FF-OCT, *Light CT scanner, LL-Tech SAS (Paris, France)*	1.5 × 1.0	> 1	NS	100	Ex vivo	Resected specimens	2 Pathologists	72	74	
Oesophageal cancer	Zuccaro et al. (2001)	OCT, *manufacturer not specified*	12 × 20	1	3	138	In vivo (endoscopic)	AC	23 Individuals	–	–	95
	Hatta et al. (2010)	OCT, *Light Lab Imaging (Boston, Mass) and HOYA (Tokyo, Japan)*	11 × 30	1.5	NS	144	In vivo (endoscopic)	SCC	1 Gastroenterologist	–	–	93
	Hatta et al. (2012)	OCT, *Light Lab Imaging (Boston, Mass) and HOYA (Tokyo, Japan)*	11 × 30	1.5	NS	131	In vivo (endoscopic)	SCC	1 Gastroenterologist	–	–	95
Colorectal cancer	Ashok et al. (2013)	(Fourier Domain) OCT, *University of Edinburgh (Edinburgh, UK)*	6.2 × 17	1.2	5	62	Ex vivo	Resected specimens	Computer	78	74	–
Prostate cancer	Dangle et al. (2009)	OCT, *Niris™ System, Imalux Corporation (Cleveland, OH, USA)*	10–20 × 10–20	2–3	1.5	100	Ex vivo	Resection margin	NS	70	84	–
	Lopater et al. (2016)	FF-OCT, *Light CT scanner, LL-Tech SAS (Paris, France)*	1.5 × 1.5	> 1	Mean: 261	119	Ex vivo	Biopsies	3 Pathologists	63	74	–
Renal cancer	Lee et al. (2012a, b)	OCT, *manufacturer not specified*	4 × 14	NS	NS	35	Ex vivo	Resected specimens	Three observers	96	96	–
	Jain et al. (2015)	FF-OCT, *Light CT scanner, LL-Tech SAS (Paris, France)*	1.5 × 0.8	NS	NS	67	Ex vivo	Resected specimens	1 Uropathologist	100	100	–
	Wagstaff et al. (2016)	OCT, *Ilumien™ Optis™, St. Jude Medical (Saint Paul, MN, USA)*	15 × 20	NS	NS	40	Ex vivo	Renal biopsies	Computer	86	75	–
Bladder cancer	Manyak et al. (2005)	OCT, *manufacturer not specified*	10 × 15	1	1.5	87	Ex vivo	Biopsies	1 Reviewer	100	89	–
	Hermes et al. (2008)	OCT, *Aachen University (based on Sirius 713, Heidelberg Engineering GmbH, Lübeck, Germany)*	3 × 10	NS	4–16	142	Ex vivo	Resected specimens	1 Reviewer	84	78	–
	Goh et al. (2008)	OCT, *Niris Imaging System (Imalux, Cleveland, OH)*	10 × 20	1–2	1.5	94	In vivo	Biopsies and resected specimens	1 Surgeon	100	90	–
	Ren et al. (2009)	OCT, *Stony Brook University, (New York, USA)*	10 × 10	2.1	8 frames/s	110	In vivo	Biopsies	Urologists/OCT researchers	94	81	–
	Karl et al. (2010)	OCT, *Niris Imaging System (Imalux, Cleveland, OH)*	10 × 20	1–2	1.5	102	In vivo	biopsies	NS	100	65	–
	Gladkova et al. (2011)	Cross-polarization OCT, *Institute of Applied Physics of the Russian Academy of Sciences (Nizhny Novgorod, Russia)*	15 × 25	NS	2	360	Ex vivo	Biopsies	7 reviewers	94	84	–
	Montagne et al. (2017)	FF-OCT, *Light CT scanner, LL-Tech SAS (Paris, France)*	1.5 × 1.0	> 1	NS	24	Ex vivo	Resected specimens	2 unexperienced reviewers; 1 FF-OCT expert	Unexperienced: 93 Expert:100	Unexperienced: 78 Expert: 89	–
Ovarian cancer	Nandy et al. (2016)	FF-OCT, *manufacturer not specified*	1.6 × 3.9	NS	NS	56	Ex vivo	Resected specimens	Computer: logistic classifier model	92	88	–

*OCT* optical coherence tomography, *NS* not specified, *HD-OCT* high definition optical coherence tomography, *SCC* squamous cell carcinoma, *AC* adenocarcinoma, *PDC* poorly differentiated carcinoma, *FF-OCT* full-field optical coherence tomography

### Skin cancer

Skin tumors are usually divided into melanoma and non-melanoma cancer. Because of its aggressive character, the only curative treatment for local melanomas is surgical resection in combination with sentinel lymph node mapping. However, in the last years novel target therapies were developed which showed great potential in patients with unresectable or metastatic melanoma (Tripp et al. 2016). For basal cell carcinoma (BCC), which is a non-melanoma cancer and the most common type of cancer in caucasians worldwide, many treatment options are available and applied, dependent on the tumor characteristics and patient’s preference (Verkouteren et al. 2017). Mohs micrographic surgery is currently performed in many clinics, to obtain free resection margins. Nevertheless, it would be preferable for both the patient and surgeon to obtain real-time feedback of the margin involvement during surgery. Many studies have determined the capacity of OCT for visualization of different types of skin cancer.

### Malignant melanoma

**Fig. 2:**
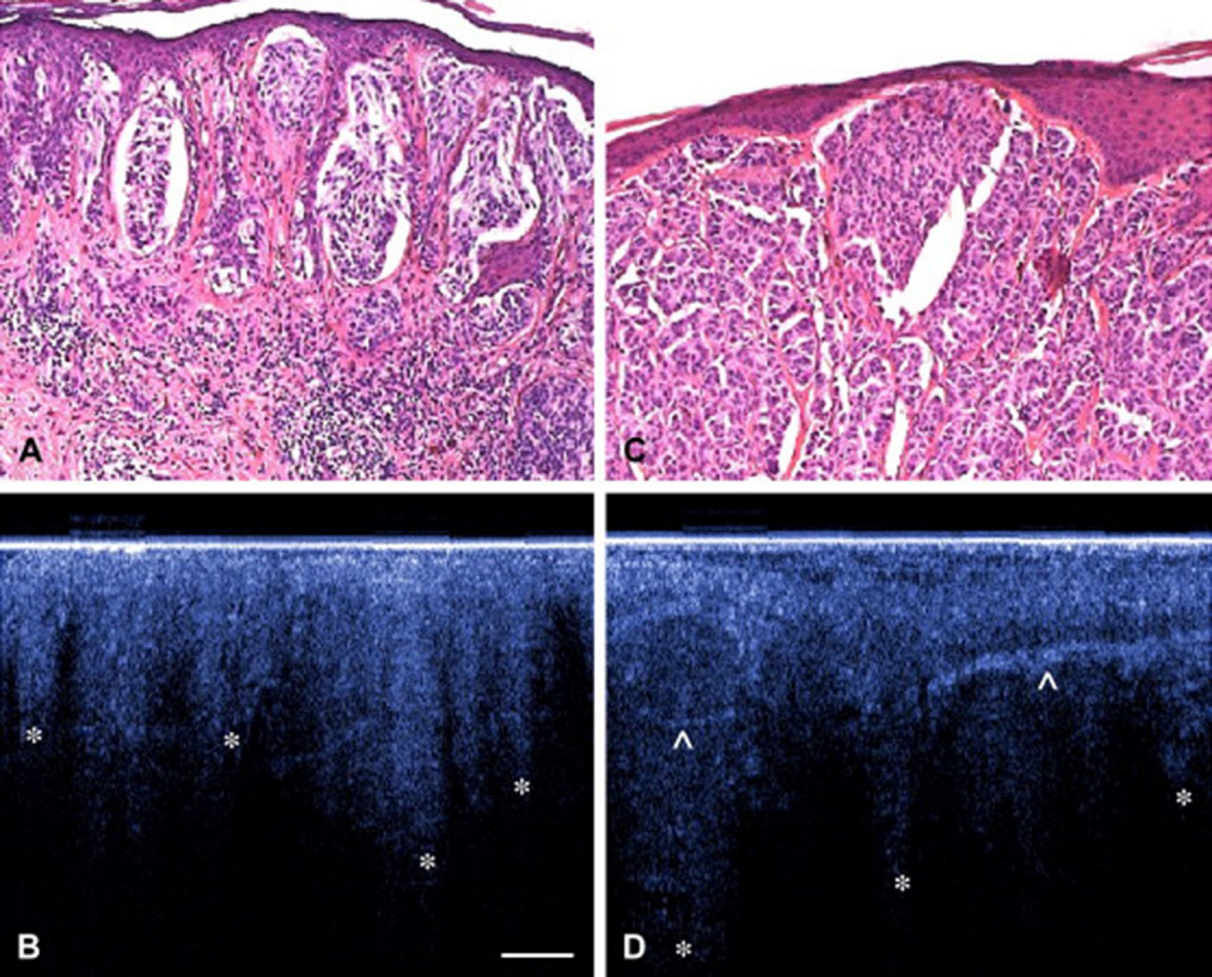
Example of corresponding OCT and histology images of two melanomas Upper panel (**a**, **c**): Hematoxylin and eosin (H&E) images of a superficial spreading melanoma. Lower panel (**b**, **d**): OCT images of distorted skin architecture, including large vertically arranged icicle-shaped structures (*). Prominent hyperreflective structures are corresponding to dense collagen cords of encapsulated tumor lobules. Reprinted by permission from Elsevier: Journal of the American Academy of Dermatology (Gambichler et al. 2007). © 2007

OCT images of a malignant melanoma showed irregular structures in the lower epidermis, which corresponded to histology. The basement membrane zone was also not visible, which made these characteristics specific for malignant melanoma (Welzel et al. 1997). Moreover, other characteristics have been investigated. In general, two characteristics were often visible: (1) the presence of horizontal highly reflective cords in the epidermis and dermis, which probably correspond to dense collagen cords of encapsulated tumor lobules and (2) the presence of large vertical icicle-shaped structures reaching the reticular dermis with the peak aspect, which corresponded to tumor cells and lymphocytes infiltration on histology (Fig. 2) (Boone et al. 2014; Gambichler et al. 2007, 2014).

### Basal cell carcinoma

Several specific features for BCC were suggested, of which disruption of layering, hyporeflective rounded areas surrounded by a hyperreflective halo (honeycomb structure), peripheral palisading and dilated vessels, well circumscribed black/signal poor areas were the most common and characteristic (Alawi et al. 2013; Bechara et al. 2004; Boone et al. 2012; Coleman et al. 2013; Forsea et al. 2010; Gambichler et al. 2007, 2014; Hinz et al. 2012; Jorgensen et al. 2008; Khandwala et al. 2010; Maier et al. 2013; Meekings et al. 2016; Mogensen et al. 2009a, b, 2011; Olmedo et al. 2006, 2007; Pomerantz et al. 2011; Wang et al. 2011). Two studies evaluated the diagnostic accuracy of OCT in detecting basal cell carcinomas in vivo, showing good results with sensitivity and specificity ranging from 79 to 94 and 85–96%, respectively (Jorgensen et al. 2008; Mogensen et al. 2009a). Ulrich et al. evaluated the diagnostic value of OCT combined with clinical and dermoscopic assessment with sensitivity and specificity of 96 and 75%, respectively, which resulted in a higher diagnostic accuracy compared to clinical and dermoscopic information (Ulrich et al. 2015). However, even for experienced observers, it was difficult to distinguish BCC from actinic keratosis, which was illustrated by the 50% error rate (Mogensen et al. 2009a). Differentiation between the several BCC subtypes was difficult; however, variants of OCT like HD-OCT and multi-beam swept source OCT (MSS-OCT) showed potential for clinical use (Boone et al. 2012; Gambichler et al. 2007; Meekings et al. 2016). Moreover, many studies were performed to assess the surgical margins during Mohs surgery (Alawi et al. 2013; Coleman et al. 2014; Cunha et al. 2011; Durkin et al. 2014; Iftimia et al. 2016; Maier et al. 2014; Pelosini et al. 2013; Pomerantz et al. 2011; Wang et al. 2013). Conventional OCT yielded to a sensitivity of 19% and specificity of 56%, whereas HD-OCT showed an improved sensitivity of 75% and a specificity of 64% (Cunha et al. 2011; Maier et al. 2014).

### Conclusion

Diverse specific tumor characteristics for both melanoma and BCC were composed. In case of melanoma, no diagnostic studies were performed with OCT. OCT showed good results in BCC detection; however, margin assessment, which is clinically most relevant, was much more difficult even with higher resolution OCT.

### Oral cancer

Oral cancer, of which squamous cell carcinoma accounts for 90% of the cases, is often treated by a combination of surgery and radiotherapy (Neville and Day 2002). Due to the difficult location and the surrounding vital structures, it is of outmost important to achieve complete tumor removal. OCT was utilised in ten studies to evaluate its potential use.

### Oral (pre-)cancerous lesions

Several parameters were important to distinguish between benign and (pre)malignant oral lesions, such as disorganization of epithelial stratification (irregular collagen vessels), epithelial and/or keratin thickening, micro-structure invasion, heterogeneous cell distribution, and disorganization of the basement membrane (Hamdoon et al. 2016; Leeuw et al. 2015; Wilder-Smith et al. 2009). Four studies in 19–125 patients showed that dysplasia detection was possible both after training of independent reviewers and using quantitative analysis (Adegun et al. 2012; Hamdoon et al. 2013; Jerjes et al. 2010; Lee et al. 2012a). Computer analysis, using a 70% standard deviation of the epithelial thickness, yielded a sensitivity of 82% and a 90% specificity, which indicated that epithelial thickness is one of the most characterizing features of oral dysplasia (Hamdoon et al. 2012; Lee et al. 2012a). Squamous cell carcinoma was very well identified, resulting in a sensitivity of 82% and 93% and a specificity of 87 and 93%, as demonstrated by Wilder-Smith et al. (2009) and Hamdoon et al. (2016). De Leeuw et al. (2015) evaluated 57 FF-OCT images for the presence of cancerous lesions, yielding a 85% accuracy for reviewer 1 and a 89% accuracy for reviewer 2. In both studies, image assessment was performed by two independent reviewers, who used the main features of malignancy on OCT images. Using a variant of OCT, Swept Source OCT (SS-OCT), Tsai et al. performed quantitative analysis and showed that in premalignant tissue, the epithelium became significantly thicker and the standard deviation became larger, due to epithelial disorganization (Tsai et al. 2008, 2009).

### Conclusion

Several characteristics for (pre)malignant oral tissue were proposed, all showing good diagnostic accuracies. These morphological characteristics were confirmed by quantitative analysis; nevertheless, no intraoperative studies were yet performed for real-time evaluation of the surgical resection margins.

### Lung cancer

The diagnosis of lung cancer, which is the most common cancer in men worldwide, is often made by CT and flexible bronchoscopy. However, bronchoscopy lacks sensitivity, especially for early stage malignancies (Andolfi et al. 2016). Five studies utilized OCT as an additional imaging tool for visualization of lung cancer both during bronchoscopy and after surgery on resected specimens.

### OCT during bronchoscopy

Bronchial malignancies were generally characterized on OCT images by a thickened epithelium wall and loss of subepithelial identifiable microstructures. Tumor invasion was visible as a disappearance and/or disturbed architecture of the basement membrane (Lam et al. 2008; Michel et al. 2010; Whiteman et al. 2006). In normal lung tissue, the basement membrane and the lamina propria were visualized as highly reflective layers, due to the presence of collagen vessels. Deeper layers containing seromucinous glands, connective tissue, and cartilage, were characterized by polymorphic light and dark areas (Lam et al. 2008; Michel et al. 2010). Hariri et al. composed specific OCT criteria for the different tumor types: adenocarcinoma, squamous cell carcinoma, and poorly differentiated carcinoma (Hariri et al. 2015). Adenocarcinomas were characterized by round or angulated signal-poor to signal void structures, which were typically small and secondly by lack of signal-intense (bright) nests. Squamous cell carcinoma could be recognized by the presence of signal intense nest (brighter than surrounding tissue), which were round or irregularly shaped. These nests may have variably sizes, and sometimes, also areas of necrosis were visible as signal-poor areas. Lack of round/angulated signal-poor structures and lack of signal-intense nests were specific for poorly differentiated carcinomas.

These criteria were applied by Hariri et al. in a prospective validation cohort, in which three readers evaluated 153 OCT images acquired from five patients, divided over two assessments (separated by 7 months) preceded by a training session (Hariri et al. 2015). The overall accuracy improved from 81.8 to 83.3% after the second assessment.

### Surgical resected specimens

FF-OCT provided high-resolution images of both normal and malignant resected lung specimens up to a depth of 5–15 µm. Normal lung tissue was recognized by the typical leace-like pattern, which was formed by the alveoli and their septal walls, visible as signal-void dark areas and bright areas, respectively. Other lung components could also be identified, such as the pleura (bright signal), blood vessels, and bronchi (dull grey signal). Adenocarcinomas, characterized by their predominant lepidic growth pattern, could be really well identified. Tumor cells were also larger than normal cells, although they appeared to have a similar signal (dull grey) as normal cells (Jain et al. 2013).

### Conclusion

Although the diagnostic accuracy was fairly high and OCT during bronchoscopy seems feasible in above-mentioned studies, OCT is yet not adequate as a complete replacement for tissue biopsy. However, it has the potential to be implemented in bronchoscopy procedures for diagnosis of lung tumors. Intraoperative use for margin assessment of tumor detection is yet not evaluated.

### Breast cancer

Breast cancer, which is the most common type of cancer in women, is responsible for 14% of the cancer-related deaths annually (Siegel et al. 2018). In breast cancer surgery, it is extremely important that borders of the excised specimen do not contain any tumor cells, since these positive margins are associated with a higher risk of local recurrence of the primary tumor (Pleijhuis et al. 2009). Not only margin assessment, but also intraoperative staging by sentinel lymph node mapping is often performed in breast cancer patients (Lyman et al. 2005). Eight studies evaluated the use of OCT in resected surgical specimens and five studies evaluated the use of OCT for lymph node analysis.

### Tumor detection surgical specimens

Diverse tumor-specific criteria were developed based on histological features. Invasive ductal adenocarcinomas, which showed infiltrating tumor cells in surrounding tissue and surrounding fibrous tumor stroma, were clearly visible (Assayag et al. 2014; Yao et al. 2017; Zhou et al. 2010). Mucinous carcinomas could be recognized by mucin with floating tumor cells, which were reflected in the OCT image. Assayag et al. proposed three FF-OCT-specific criteria for malignancy in addition to macroscopic characteristics, such as the absence of normal breast tissue structures and the presence of stellate lesions: (1) the presence of adipocytes with irregular size (fat infiltration); (2) highly scattering trabecula aspect of fibrous tissue; (3) the presence of dark grey areas surrounded by white fibrous structures; (3) (Assayag et al. 2014). Especially, white fibrous structures were suspect for tumor stroma, whereas grey fibrous structures were associated with scar fibrous tissue in benign breast lobules. Furthermore, ex vivo analysis of resection margins was performed by Nguyen et al. (2009). Thirty-seven breast cancer specimens were used for analysis, divided into a training set and a study data set. Of each specimen, 5–10 images were taken, resulting in 210 images used for the study data set and pathologic analysis by one researcher. Analysis showed a sensitivity and specificity of 100 and 82%, respectively, in tumor detection compared to histology, which is the current golden standard. Feasibility of multimodal imaging, by combining OCT with ultrasound and dye-enhanced wide-field polarization imaging, was demonstrated by Patel et al. (2013) and Curatolo et al. (2012). Computer analysis showed.

**Fig. 3:**
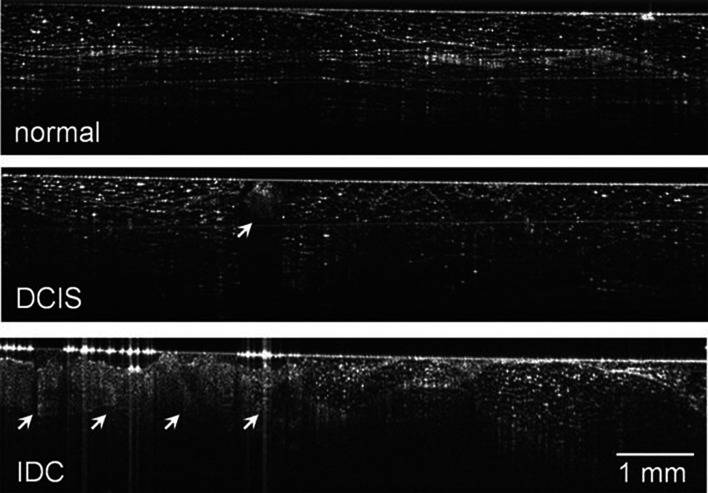
Handheld OCT during breast cancer surgery. Upper panel: normal breast tissue with well-defined boundaries, linear structures, and regular texture. Middle panel: arrow shows an example of a ductal carcinoma in situ, characterized by irregular texture and significant shadowing. Lower panel: an example of an invasive ductal carcinoma (arrows) showing regions with disturbed tissue architecture. Reprinted by permission from Springer Nature: Annals of Surgical Oncology (Zysk et al. 2015). © 2015

Recently, a handheld OCT camera has been developed, which has been used in two studies, that evaluated the diagnostic accuracy of the camera after obtaining the ex vivo final margins in 46 and 35 patients, respectively (Erickson-Bhatt et al. 2015; Zysk et al. 2015). In vivo imaging was feasible, although ex vivo images of the margins could be directly correlated to the histology slices, which consequently were used in both cohorts (Fig. 3). After comparison of histology with readers’ interpretation, they showed a diagnostic accuracy in tumor detection at the surgical margins varying between 58 and 88%. The authors stated that this variability could be explained by the minimal training preceded by image evaluation and by the experience in studying OCT images of the reader.

### Lymph nodes detection

Normal lymph nodes were characterized by a distinct capsule that was highly scattering, in comparison with the lower scattering cortex. The lymphoid follicles, which were visible as a circular texture on the OCT image, could also be clearly observed in the cortex (McLaughlin et al. 2010; Nguyen et al. 2010). Lymph node invasion was characterized by loss of normal tissue architecture, the presence of highly heterogeneous tendril-like structures, and the presence of areas with highly backscattering areas, possibly due to changes in size and texture of the cell nuclei. One study compared the diagnostic accuracy of OCT to histology, after training of three reviewers, which resulted in an overall sensitivity and specificity of 58.8 and 81.4%, respectively (Nolan et al. 2016).

To improve the capability to distinguish benign and malignant invasion of lymph nodes, parametric imaging of the local attenuation coefficient was applied in OCT images and showed promising results in two feasibility studies (McLaughlin et al. 2010; Scolaro et al. 2012). FF-OCT showed a more detailed view of the lymph nodes, of which the strong stromal reaction, caused by tumor invasion, was one of the most characterizing morphological features for lymph node invasion on FF-OCT images. FF-OCT showed an improved sensitivity of 89% and specificity of 87% compared to regular OCT after training of two independent reviewers (Grieve et al. 2016).

### Conclusion

OCT was used for both tumor detection and sentinel lymph node detection in breast cancer patients. Diverse specific criteria were applied and showed high diagnostic accuracy in margin assessment compared to histology. A handheld OCT camera, which could be used intraoperatively, was also applied for margin assessment and showed promising results. This indicates that with more training and further development, OCT could be used as an additional tool for intraoperatively tumor detection. For lymph node detection, especially, FF-OCT was able to distinguish malignant invasion of lymph nodes from benign lymph nodes with high sensitivity and specificity.

### Hepatopancreaticobiliary (HPB) tumors

The diagnosis of pancreaticobiliary cancers is often made by taking biopsies during endoscopic intervention. However, the current diagnostic accuracy for both pancreatic and biliary malignancies lacks sensitivity (Burnett et al. 2013; Chen et al. 2012). Moreover, in liver and pancreatic surgery, tumor positive resection margins defined as ≤ 1 mm tumor-to-margin distance, are found up to 24 and 75%, respectively (Are et al. 2007; Verbeke and Menon 2009). Consequently, some progress in diagnostic accuracy of HPB tumors could be made. Nine studies evaluated the use of OCT both during endoscopy and in resected specimen.

### OCT during endoscopy

OCT was used to distinguish malignant and benign pancreatic duct strictures both in vivo and ex vivo during routine endoscopic retrograde cholangiopancreatography (ERCP) procedures (Testoni et al. 2005, 2006a, b, 2007). Using disturbance of normal three-layer architecture with heterogenous backscattering as marker for the presence of tumor, *ex vivo* analysis of 100 OCT images of 10 patients showed an overall sensitivity and specificity for tumor detection of 78.6 and 88.9%, respectively (Testoni et al. 2005). Moreover, a concordance between OCT and histology for detection of a pancreatic adenocarcinoma was seen in 97.6% of the 126 images (Testoni et al. 2005). In vivo analysis resulted in a 100% accuracy for detection of neoplastic pancreatic ductal strictures (Testoni et al. 2007). Two criteria for malignant strictures were used: (1) unrecognizable layer architecture and (2) heterogeneous backscattering of signal.

Biliary duct imaging using OCT was performed in 2009 by Arvanitakis et al. (Arvanitakis et al. 2009). They used the above-mentioned criteria for detecting of malignant biliary strictures, which was accurate in 84% of the included 37 patients. OCT seemed favorable in preoperative detection of unknown biliary strictures compared to randomly taken biopsies, which resulted in a 67% accuracy in the same cohort.

Besides ERCP, endoscopic ultrasound-fine needle aspiration (EUS-FNA) is often used for taking biopsies for establishing the diagnosis of pancreatic masses. Grieve et al. (2015) evaluated the feasibility of FF-OCT in evaluation of FNA specimens acquired during EUS. Three images of pancreatic ductal adenocarcinomas (PDAC), two images of neuroendocrine pancreatic tumors, and two images of pancreatic metastases from renal cell carcinomas were included in the analyses and compared to the histology. PDAC was recognized by intense dark malignant cell clusters with irregular borders and high nuclear density. Glandular differentiation was indicated by atypical tall columnar epithelium and the presence of luminal spaces. Neuroendocrine pancreatic tumors were also easily identified by areas with neoplastic endocrine tumor cells, which appeared darker than normal pancreatic tissue. Pancreatic renal cell metastases showed a fair matching with histology. One of the two images showed good correspondence and was recognised by sheets of large cells, which compressed the vessels.

### Surgical resected specimen

Iftimia et al. (2011) used OCT for detection of several types of pancreatic cystic tumors: mucinous cystic neoplasm (MCN), intrapapillary mucinous neoplasm (IPMN), and serous cystadenoma (SCA). After developing OCT criteria for differentiating between MCNs, SCAs, and IPMNs, the investigators (a gastroenterologist, a radiologist and a pathologist) underwent training based on 20 OCT images of fresh-resected pancreatic specimens. After that, they were independently asked to evaluate 46 OCT images, resulting in a high sensitivity in distinguishing mucinous vs non-mucinous cystic lesions (95.6% for the gastroenterologist and 100% for the radiologist and pathologist).

**Fig. 4:**
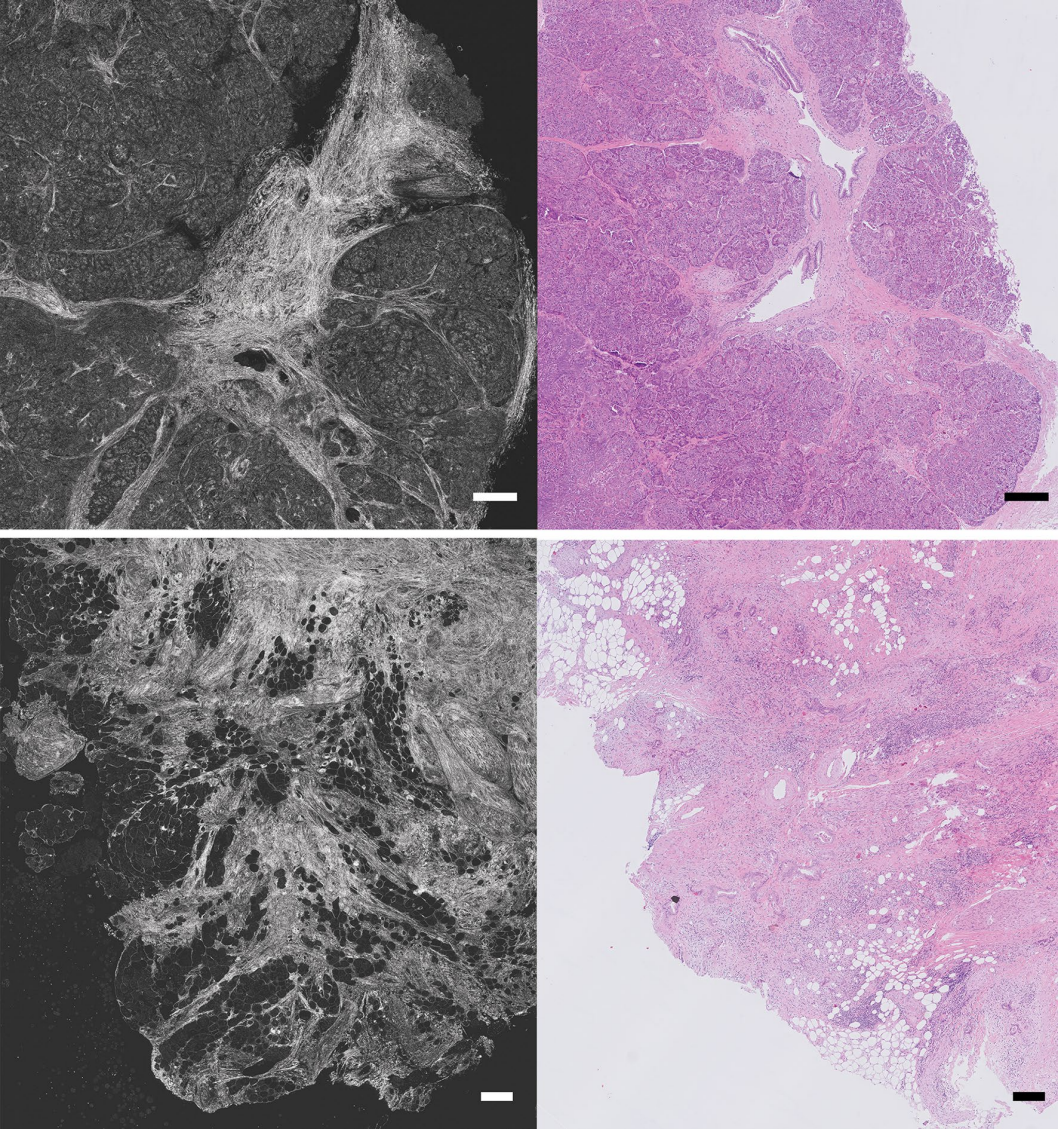
FF-OCT images of the pancreas. Upper panel: FF-OCT image and corresponding hematoxylin and eosin (H&E) image of normal pancreatic tissue. Lower panel: an example of an FF-OCT image of a moderately differentiated pancreatic adenocarcinoma with corresponding H&E image, showing tumor cells infiltrating into fat tissue (Bar = 250 µm)

Van Manen et al. (2017) evaluated the accuracy of FF-OCT in detecting pancreatic tumors in resected surgical specimens. Two pathologists were asked to evaluate 100 FF-OCT images after a training set, which resulted in a combined sensitivity and specificity of 72 and 74%, respectively, compared to histologic diagnosis. Moreover, they developed specific criteria for different types of pancreatic tumors. Especially, in case of pancreatic ductal adenocarcinoma, disorganization of glands and the presence of tumor stroma were really well visible (Fig. 4). However, due to low endogenous contrast, cell nuclei could not be visualized, whereas sometimes the collagen dominated the field of view due too much backscattering, which was mistaken for tumor stroma.

Zhu et al. (2015) evaluated the feasibility of FF-OCT in resected liver specimens. Normal liver structures, such as blood vessels, bile ducts, and sinusoidal spaces, could be really well identified. Hepatocellular carcinoma was recognized by the presence of nuclear atypia and large tumor nodules separated by thick fibrous bands.

### Conclusion

The role of OCT was evaluated both during endoscopy and on resected specimens of both cystic and solid tumors. OCT during ERCP showed high accuracy in detection of pancreatic or biliary strictures. Mucinous cystic lesions could be really well identified and distinguished from non-mucinous lesions. FF-OCT was feasible in ex vivo EUS-FNA biopsies, pancreatic, and liver specimens. Especially, in liver and pancreatic specimens, diverse tumor characteristics were found.

### Oesophageal cancer

Oesophageal cancer, one of the most lethal cancers in the western world, is usually divided in adenocarcinoma and squamous cell carcinoma (SCC) (Pennathur et al. 2013). One of the independent risk factors for an oesophageal adenocarcinoma is a Barrett’s oesophagus (BE), which is a transition of normal squamous mucosa into columnar (gastric) epithelium (= metaplasia), which could be considered as a pre-malignant stadium. Currently, most patients with BE undergo endoscopic surveillance, which is controversial. Moreover, development into dysplastic or neoplastic tissue could only be detected by taking biopsies, frequently accompanied with a sampling error (Falk et al. 1999). Thirteen studies evaluated the role of OCT during endoscopy in patients with suspected oesophageal lesions.

### Barrett’s oesophagus and adenocarcinoma

Bouma et al. performed the first in vivo study in 32 patients, who underwent routine endoscopy, and developed some characteristics of BE on OCT images (Bouma et al. 2000). Due to high scattering of the metaplastic epithelium, there was a loss of normal layered architecture. In normal oesophageal tissue, the five oesophageal wall layers (squamous epithelium, lamina propria, muscularis mucosae, submucosa, and muscularis propria) could easily be recognised by their relative difference in reflection (Bouma et al. 2000; Hatta et al. 2010; Jackle et al. 2000; Li et al. 2000). Together with the presence of inhomogeneous tissue contrast and abnormal and disorganised glands below the epithelial surface, visible as pockets of low reflectance, it is called BE. Especially, patients with BE without dysplasia or low grade dysplasia, the muscularis mucosae and submucosal layers often could be preserved (Chen et al. 2007; Cobb et al. 2010). Poneros et al. (2001) applied these criteria in a validation cohort in patients, who underwent routine upper endoscopy, which resulted in a sensitivity and specificity of 100 and 93% for BE detection, respectively. OCT was also used for detection of BE before and after radiofrequency ablation treatment. Unfortunately, in the minority of the patients (7.7%), OCT was capable to distinguish normal glands from buried Barrett’s glands (Swager et al. 2016). Another study showed a 81% sensitivity and 66% specificity in detection of BE, indicating that OCT is currently not accurate enough compared to histology (Evans et al. 2007).One study evaluated the capacity of OCT for detection of oesophagus dysplasia (Isenberg et al. 2005). Normally, dysplasia is divided in low-grade and high-grade dysplasia, which consequently results in different clinical approach, i.e., resulting in oesophageal resection or not. Dysplasia was detected on OCT by reduced light scattering and loss of tissue architecture, which are currently the only known criteria. Evaluation of 314 OCT images of 33 patients by four endoscopists resulted in a sensitivity and specificity (compared to histology of the biopsies) of 68 and 82%, respectively (Isenberg et al. 2005). However, the authors did not make any difference between low-grade dysplasia, high-grade dysplasia, or neoplasia. Chen et al. (2007) more specifically described high-grade dysplasia as the presence of irregular and distorted glandular architecture and closely packed glands.

**Fig. 5:**
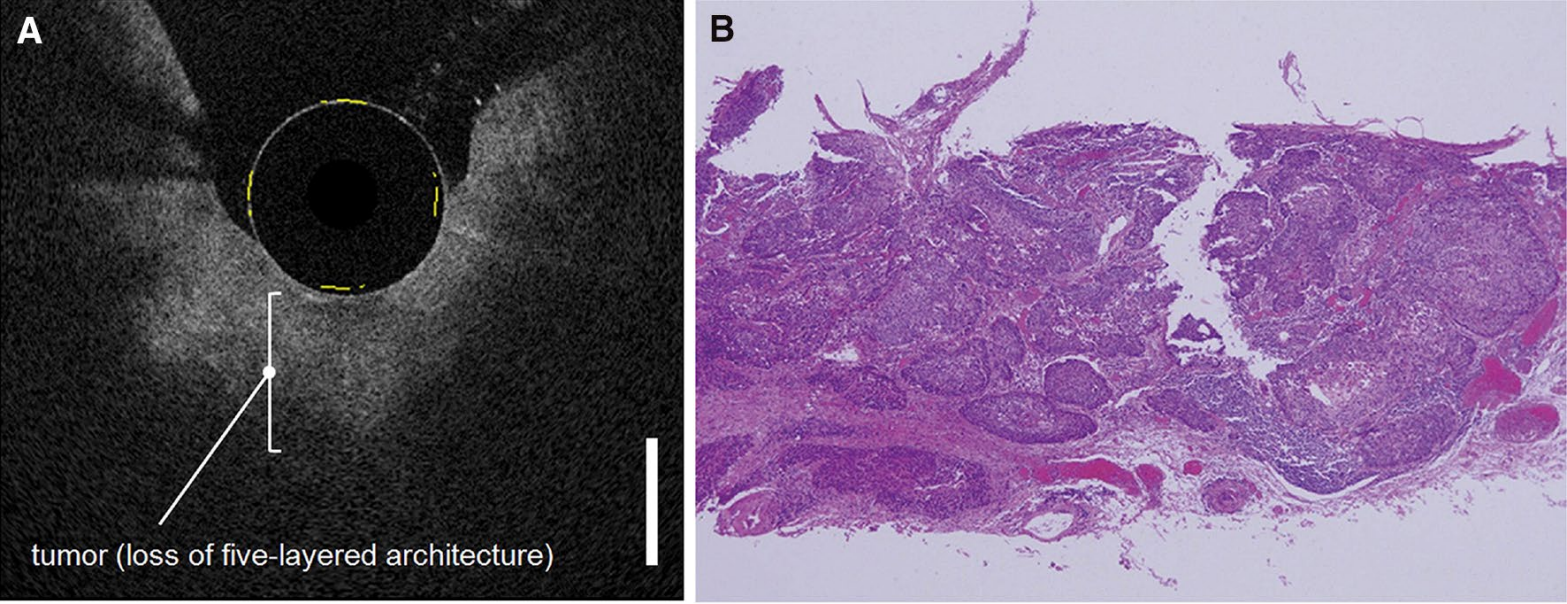
Example of endoscopic OCT of an esophageal squamous cell carcinoma. Corresponding OCT (**a**) and histology (**b**) image of tumor invasion in the submucosal layer, resulting in a loss of the five-layered architecture (Bar = 1000 µm). Reprinted by permission from Elsevier: Gastrointestinal Endoscopy (Hatta et al. 2010). © 2010

Adenocarcinomas were characterized on OCT images by a neoplastic epithelium, which contains large pockets of mucin surrounded by fibrotic and hypervascular tumor stroma (Bouma et al. 2000). Sometimes, infiltration of heterogeneous structures into the muscular layers could be seen as a feature of tumor invasion. Irregular shape and crowd of submucosal glands also advocated the presence of adenocarcinoma (Fig. 5) (Chen et al. 2007; Evans et al. 2006). Detection of adenocarcinoma in patients who underwent upper endoscopy for several reasons showed potential, with a detection rate of 95% (Zuccaro et al. 2001).

### Squamous cell carcinoma

Hatta et al. (2010) compared the capability of OCT for SCC invasion detection in the different layers of the oesophagus to histology. Superficial invasion into the epithelium layer was difficult to distinguish from normal oesophagus tissue. However, the researchers were able to detect the tumor invasion level with a high overall accuracy: 92.7%. Furthermore, they compared the diagnostic accuracy of OCT with high-frequency EUS in a larger cohort of 123 patients (Hatta et al. 2012). OCT showed a significantly higher accuracy than high-frequency EUS (90.1 vs 77.1%; *P* = 0.0046).

### Conclusion

OCT has been evaluated for establishing the diagnosis of oesophageal SCC, BE, and adenocarcinoma. Diverse characteristics of BE, dysplasia, adenocarcinoma, and SCC were applied, which showed promising results. However, differentiating between normal glands and Barrett’s and tumor glands was really difficult.

### Colon cancer

Screening for colon cancer is currently mainly performed using fecal occult blood testing, in suspected cases followed by endoscopy (Ali et al. 2003). Adenomatous polyps, which carry risk of cancer development, are often found during colonoscopy. They can macroscopically be divided based on their growth pattern into tubular and villous polyps and microscopically they are generally classified as low-grade or high-grade dysplasia. Currently, no studies have described the use of OCT for cancer detection; however, six studies assessed the use of endoscopic OCT as a screening tool for premalignant polyp detection.

### Detection of (pre)malignant tissue

Tearney et al. (1997b) first described the feasibility of OCT during colonoscopy for premalignant tissue detection. Normal colonic wall features were described by Westphal et al. (2005). The mucosa was visible as a reflective layer, including an underlying thin hyperreflective line, whereas the submucosa appeared highly variable probably caused by compression related to the colonoscopy procedure. Pfau et al. (2003) described the different features visible on OCT concerning the most common forms of polyps and showed that adenomatous polyps were significantly more disorganized and more hypo-reflective than hyperplastic polyps. Colorectal neoplasms were described as polyps with an uneven surface. On OCT, areas of malignant cells appeared bright, whereas non-cellular regions were less reflecting (Jackle et al. 2000). Quantitative analysis was performed for colorectal cancer in two studies, which showed that malignant tissue has lower scattering properties and less variation of signal intensity from the surface yielding a 78% sensitivity and 74% specificity (Ashok et al. 2013; Zhang et al. 2012).

### Conclusion

The use of OCT during colonoscopy for tumor detection has not been frequently analysed. Currently, presumably quantitative analysis could be performed for both tumor and polyp detection; however, the diagnostic accuracy lacks sensitivity.

### Urological cancer

Most of the time surgery is not the first choice of treatment for urological cancers.. Establishing the correct diagnosis before chemoradiotherapy is of outmost important. Therefore, OCT has been mainly used as an additional tool during biopsy taking in both five studies for prostate, six studies for renal and seven studies for bladder cancer detection, respectively.

### Prostate cancer

D’Amico et al. (2000) first described the feasibility of OCT for detection of tumor in prostate biopsies. The first large cohort study, performed by Dangle et al. (2009) *p* included 100 samples of which 10 had positive surgical margins. Using homogeneity of the prostate epithelium and the presence of prostatic stroma as assessment criteria, OCT yielded a sensitivity of 70% and specificity of 84%. More detailed information about malignant features were visualized using FF-OCT, such as the presence of irregular gland contours, infiltration of variably sized glands between benign glands, and the presence of cribriform patterns (Lopater et al. 2016). These characteristics were used during analysis by three independent pathologists, resulting in a 63% sensitivity and 74% specificity. However, a learning curve was observed, leading to a diagnostic accuracy of 82% at the end of the assessment. More recently, quantitative analysis of prostate biopsies showed significant differences in attenuation coefficient between normal and malign prostate tissue (Muller et al. 2016, 2017).

### Renal cancer

Linehan et al. performed the first ex vivo study on 20 resected renal specimens (Linehan et al. 2011). In general, in malignant tissue, there was a lack of normal structures; however, in some cases, tumor samples had heterogeneous areas, which mimicked normal kidney tissue. Furthermore, high sensitivities and specificities (up to 100%) for renal tumor detection were achieved in another study by Lee et al. (2012b). Three pathologists evaluated 35 OCT images of both normal and tumor resected specimens. Renal tumors were characterised by increased scattering tumor nests, which were separated by hyposcattering stroma (Lee et al. 2012b). More specific, distinguishing renal cell carcinoma and renal oncocytoma (benign renal tumor) was possible by both morphological characteristics and after quantitative analysis of the attenuation coefficient (Barwari et al. 2012; Lee et al. 2012b; Wagstaff et al. 2016). Computer analysis also showed the attenuation coefficient of normal renal parenchyma to be significantly differed from malignant tumors (Barwari et al. 2011, 2012). Moreover, several signatures of different kidney tumors could be recognized as described by Jain et al. (2015). After training, the uropathologist was able to distinguish benign and malignant tumors (67 FF-OCT images) with a 100% accuracy and achieved a 80% accuracy for correct subtyping of these tumors.

### Bladder cancer

Seven studies evaluated the diagnostic value of OCT for the detection of cancer in bladder biopsies or resected specimens in populations varying between 21 and 116 patients (Gladkova et al. 2011; Goh et al. 2008; Hermes et al. 2008; Karl et al. 2010; Manyak et al. 2005; Montagne et al. 2017; Ren et al. 2009). Disorganized tissue layers and sub-epithelial nests of tumor cells were often found in tumorous biopsies. Using these criteria, sensitivities between 84 and 100%, and specificities between 65 and 89% for tumor detection could be achieved. Using FF-OCT, more details of tumor cells, such as the presence of large nuclei and newly formed blood vessels, appearing as small bright spots, could be visualized. After training of the reviewers, a diagnostic accuracy up to 96% could be obtained, as demonstrated by Montagne et al. (2017).

### Conclusion

OCT was capable to distinguish urological tumors from benign tissue with varying accuracies. For quantitative analysis, the attenuation coefficient seems the most informative parameter.

### Ovarian cancer

Ovarian cancer is a disease with a dreadful prognosis, making it the most lethal gynaecological malignancy (Siegel et al. 2018). Treatment mainly consists of surgery, especially in early-stage disease. Eight studies, evaluated the use of OCT for detection of ovarian cancer, of which two during surgery.

### Resected specimen

Two studies evaluated the tumor characteristics based on qualitative analysis using OCT and FF-OCT, respectively (Peters et al. 2016; Yang et al. 2011a). Ovarian malignancies were characterized by the presence of hyperintense regions with irregular patterns, which turned out to be disorganised collagen fibers. Ovarian metastases could also be detected as shown by Stegehuis et al. (Peters et al. 2016). Metastatic tumor cells appeared light grey in a web-like organisation, resulting in a distorted ovarian cortex architecture. Furthermore, other studies analysed tumor images quantitatively, focussing on optical coefficients, which were derived from normalized histograms (Nandy et al. 2016; St-Pierre et al. 2017; Yang et al. 2011b, 2012). After building a logistic classifier model, Nandy et al. (2016) were able to achieve a 91.6% sensitivity and a 87.7% specificity for tumor detection based on FF-OCT images.

### Laparoscopic surgery

Boppart et al. (1999) first described the use of OCT, integrated in a laparoscopic system, in one resected ovary specimen, containing a serous papillary cystadenocarcinoma. The presence of cysts, which are surrounding by papillary structures, were characterizing for this tumor type. In vivo application of laparoscopic OCT was performed by Hariri et al. in 30 ovaries of 17 patients, who underwent surgical resection (Hariri et al. 2009). They described different features of ovarian tumors, such as papillary serous cystadenoma or adenocarcinoma. An adenoma was characterized by large, simple cystic structure with a sharp, regular, well-demarcated cyst lining. Complex, multicystic signal void structures surrounding by tumor stroma, which have a more heterogeneous signal intensity than normal stroma, were suspect for adenocarcinomas.

### Conclusion

Several subtypes of ovarian tumors could be recognized using OCT both *ex vivo* and during laparoscopic surgery. However, until today no large patient cohort studies have been reported.

## General conclusion and future perspectives

The introduction of OCT shows new opportunities during oncologic interventions. In this review, the role of OCT was evaluated for different tumor types. We have shown that several characteristics for both normal tissues and the different tumor types were established. In case of Barrett’s oesophagus, even metaplasia could be detected. OCT could be integrated during bronchoscopic and endoscopic procedures. However, the diagnostic value of OCT was evaluated in limited studies, of which an overview is provided in Table 1. In general, it can be concluded, based on this overview, that use of OCT for guiding of biopsy location during bronchoscopy, intraoperative margin assessment in case of breast cancer surgery and for visualization of pancreatico-biliary strictures during ERCP might be useful. Especially, in case of breast cancer, a developed handheld device showed promising diagnostic accuracy, which could be valuable during surgical intervention.

The great advantage of OCT is its noninvasive, reproducible and well-tolerated characteristics for in human use. However, some drawbacks could be mentioned. First of all, OCT is a relatively new modality, which needs extensive training and setting-up new clinically relevant features by building extensive atlases for instance, before adopting into clinical practice. That also states the need for automatic feature extraction and even automatic tumor detection, which was recently performed by Marvdashti et al. (2016) who integrated an automatic classifier based on known characteristics and new features, resulting in a high diagnostic accuracy of 95.4%. However, due to the availability of several OCT systems classification is difficult, although matching of optical properties is possible (Liu et al. 2017). Standardization for signal intensities for each device, which has been done after the introduction of CT devices using Hounsfield units, would make analysis easier (Davis et al. 2018). Secondly, the imaging depth is restricted up to maximum 1–2 mm in OCT and to less than 1 mm in its high resolution variants (HD-OCT, FF-OCT). Conventional OCT with resolution between 7 and 10 µm showed less microscopic details compared to the current gold standard, histology. Nevertheless, further developing of new variants of conventional OCT with increased resolution, such as FF-OCT, could improve the quality of the images, which make tumor detection feasible into the clinical practice. However, even with FF-OCT, which provides images with resolution comparable to histology, the endogenous contrast of for instance cell nuclei is not good enough for visualization. Techniques to improve the subcellular contrast, such as dynamic FF-OCT, are being developed, enhancing both structural and functional information through the detection of the intracellular activity (Apelian et al. 2016). Recently, also a handheld FF-OCT rigid endoscope has been developed, with a faster and more sensitive camera integrated than current FF-OCT systems, which paves the way for intraoperative use (Benoit a la Guillaume et al. 2016). Finally, sometimes, signal of highly backscattering tissues, such as collagen vessels or tumor stroma, disturbed the OCT images and made distinguishing benign and malignant tissue from each other complicated. On the other hand, it was shown that stroma alignment is significantly different between benign and malignant tissue for pancreatic cancer (Drifka et al. 2015) and breast cancer (Bredfeldt et al. 2014), and could, therefore, contribute to better detection of tumors on OCT images. Such information can be possibly obtained using polarization-sensitive OCT, which in addition to tissue microarchitecture can provide images of tissue birefringence (Marvdashti et al. 2016). Hariri et al. showed that Polarization Sensitive OCT (PS-OCT) has a potential to help in differentiation between lung tumor and fibrosis and Kiseleva et al. used PS-OCT to diagnose mucosal pathologies in in-vivo human bladders (Hariri et al. 2013; Kiseleva et al. 2015). In addition, other functional OCT variants, such as Doppler-OCT that visualizes tissue vasculature and spectroscopic OCT that differentiates tissue types based on signal attenuation, as well as multimodality approaches are currently a topic of research (Barwari et al. 2011, 2012; Mavadia et al. 2012; Welge and Barton 2017).

In conclusion, OCT showed promising results in tumor detection and with the development of novel probes allowing integration in bronchoscopy, flexible or rigid endoscopy, needles (Lorenser et al. 2013), handheld cameras and tethered capsules (Gora et al. 2013; Liang et al. 2016) could add important value during both preoperative diagnosis as well for intraoperative use in obtaining tumor free resection margins in the nearby future.
